# The Influence of Smartphones on Adolescent Sleep: A Systematic Literature Review

**DOI:** 10.3390/nursrep13020054

**Published:** 2023-04-07

**Authors:** Sofia de Sá, Ana Baião, Helena Marques, Maria do Céu Marques, Maria José Reis, Sandra Dias, Marta Catarino

**Affiliations:** 1 Baixo Alentejo Local Health Unit, Public Business Entity, 7800-309 Beja, Portugal; 2 Health Department, Polytechnic Institute of Beja, 7800-111 Beja, Portugal; 3 Nursing Department, University of Évora, 7000-869 Évora, Portugal; 4 Comprehensive Health Research Centre, 1150-082 Lisboa, Portugal; 5 Algarve University Hospital Center, 8000-386 Faro, Portugal; 6 Institute of Health Sciences (ICS), Universidade Católica Portuguesa, 1649-023 Lisboa, Portugal; 7 Center for Interdisciplinary Research in Health (CIIS), Institute of Health Sciences (ICS), Universidade Católica Portuguesa, 1649-023 Lisboa, Portugal

**Keywords:** smartphone, children, well-being, sleep, teenagers, media

## Abstract

(1) Background: Sleep is considered to be a complex condition for human beings, with the aim of ensuring physical and psychological recovery. Technology, including the cell phone, is a tool for teenagers that ensures they are always available to interact, even at night. This study aims to understand the influence of the use of smartphones on adolescent sleep quality. (2) Methods: The guidelines proposed by the Joanna Briggs Institute were followed. The search was conducted in October 2022 through the EBSCOhost platform, with access to the CINAHL Complete and Medline databases and through the b-On database. (3) Results: The use of electronic equipment plays an important role in adolescents’ lives. There is a negative relationship between the use of electronic equipment, such as smartphones, and sleep, for reducing both the quality and quantity of sleep. There is also a relationship between nighttime smartphone use, insufficient sleep, and mental health problems. (4) Conclusions: The use of new technologies at night causes a change in the behavior of adolescents with repercussions in terms of the quality of sleep and sleep duration and consequent well-being and performance during the day.

## 1. Introduction

Sleep is considered to be a very complex condition that serves to restructure all functions of an organism and to ensure the physical and psychic recovery of human beings. It is believed that during the sleep period, cell renewal, production of hormones and antibodies, protein synthesis, and metabolic regulation take place, which contribute in a very significant way to the physical growth of children [1].

In a questionnaire conducted in 2018 in Portugal regarding the sleep of Portuguese adolescents, it was concluded that 86.3% (N = 3894) reported difficulty in waking up in the morning and 59.7% (N = 3899) believed they sleep poorly [2]. Throughout growth, the sleep pattern undergoes changes, given that in adolescence, there is a shift in sleep patterns associated with deep changes that occur at a biological, psychological, and sociocultural level. As Bartel affirms: “The decline in adolescent sleep quantity and quality is multifactorial, and is influenced by biological, environmental, societal, and behavioral facts” [2] (p. 498).

The circadian rhythm of adolescents is bound to the inherent alterations in this growth stage. The sleep–wake cycle is regulated by melatonin, the hormone responsible for the onset of sleep, beginning its production at dusk. In adolescence, the production of this hormone occurs later, which implies that the adolescent will feel sleepy later and this sensation will last until later, coinciding with the waking hour [3].

As it is expected that changes inherent to this stage of growth will occur in adolescents’ sleep patterns, it is important to take into consideration that “Adolescence is a sensitive age period for changes in sleep-wake patterns” [4] (p. 277) and that the National Sleep Foundation [5] recommends that an adolescent should sleep between 8 and 10 h. It is also proven that the quantity of sleep hours is not always associated with quality sleep.

The World Sleep Society [6] refers to the existence of studies suggesting that the quality of sleep holds a bigger impact than its quantity, both in terms of quality of life and daily period, such that poor-quality sleep combined with precarious health are closely related to a decrease in quality of life and happiness. The World Sleep Society highlights, in turn, that sleep centers around the world invite the teaching of good “sleep hygiene,” advocating for the conservation of high-quality sleep in sufficient quantity. It also emphasizes that sleep is a basic human necessity as important as eating and drinking and indispensable for health and well-being. Good sleep along with a balanced diet and adequate physical exercise are important factors and some of the pillars of health. Finally, it states that sleep problems represent a global pandemic that puts at risk the health and quality of life of approximately 45% of the world’s population, and in this sense, an adequate understanding of this problem will help to reduce the impact of sleep disturbances on society.

For good-quality sleep, three elements are considered: duration, continuity, and depth [6]. The World Sleep Society [6], through the document “Ten commandments for better sleep”, which was released in the 2016 to celebrate the World Sleep Day, reveals the essential rules of proper sleep hygiene to maintain or restore deep, healthy, restful, and natural sleep, and underlines environmental conditions and the usage of electronic devices as a set of factors that influence the quantity and quality of sleep and, consequently, general well-being, which depends on high-quality sleep.

Nowadays, technology is constantly present in our life, and smartphones provide constant opportunities to connect with others [3], creating the need to be frequently connectable and connected to various social and sharing networks. This need is also felt among adolescents, who have, in technology, a tool that makes them always available for interaction [3]. This leads to a delay in the onset of sleep, as well as its frequent interruption through new technologies, namely the smartphone, as they coexist with the presence of noise and several standby lights, which disturb sleep [7].

This permanent connectivity that technological devices allow for increasingly presents itself as a risk factor for good-quality sleep among adolescents. Various studies confirm that “Sleep disturbance and adverse health outcomes related to screen media practices are on the rise affecting physical, cognitive, and behavioral health out-comes” [8] (p. 292).

The Portuguese Association of Sleep [7] demonstrates that, in Portugal, children and adolescents sleep, in general, for less time than is recommended, linking changes in the lifestyles of parents and children with alterations in sleep habit patterns.

Considering this topic as a current issue, since the quality of sleep is of extreme importance for good physical and intellectual development of adolescents, it was determined that this topic is of the utmost importance. The following question was formulated: “Does the use of smartphones affect adolescents’ sleep?” Therefore, the objective of this review is to understand how the use of smartphones interferes with the quality of adolescents’ sleep.

## 2. Materials and Methods

A Systematic Literature Review was performed, based on the methodological procedures defined by the Joanna Briggs Institute, and it was registered on PROSPERO platform (registration number CRD42023395696).

Using the PICO method (P—problem/population/participants (adolescents); I—intervention (use of smartphones); C—comparison/context (society); O—outcomes, results (influence in sleep)), the question for this review was formulated: “Does the use of smartphones affect adolescents’ sleep?”

The search of articles was carried out on the platform EBSCOhost, with access to the databases CINAHL Complete and Medline, also on the database b-ON, following the Health Sciences Descriptors (DeCS) [9]. As keywords, the words “smartphone”, “children”, “well-being”, “sleep”, “teenagers”, and “media” were selected, using the operator Boolean “AND” to combine the search terms. The research was conducted in October 2022 by two researchers simultaneously, applying the inclusion and exclusion criteria. A third researcher participated in the selection of studies for final review, confirming the selection and exclusion criteria, after individual reviews and researchers’ meetings. For this review, inclusion criteria were defined: randomized controlled studies, experimental studies without randomization, cohort studies and controlled cases, observational studies without group control, case series and clinical trials, with published full text, with relevance to the identified problem, written in Portuguese, English or Spanish, and with publication date between 2014 and 2022. The review considered studies that included adolescents, according to the definitions of American Academy of Pediatrics, which considers all individuals from 11 to 21 years old.

The articles whose topic was not relevant for the review in question were excluded, and those that did not address the previously defined population and Systematic Literature Reviews.

**Figure 1:**
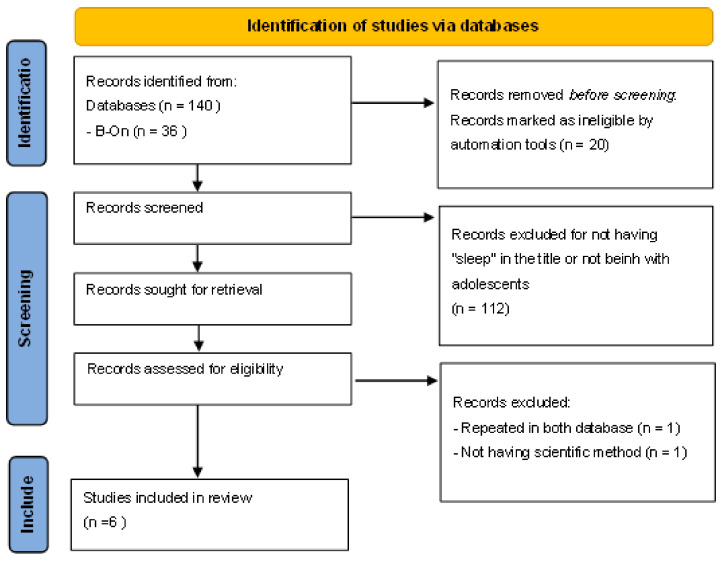
PRISMA of study selection and inclusion process [10].

Among the databases used, in total, 140 were obtained. The relevance of these articles was analyzed by reading the title, summary, and, whenever necessary, full-text reading. After applying the inclusion and exclusion criteria, 6 articles were selected, as illustrated in Figure 1.

After the selection of studies, they were evaluated critically by applying assessment grids of the level of evidence, reliability, and relevance, which allowed us to classify the studies found (Table 1).

To assess the level of evidence, the evaluation tools of Joanna Briggs Institute (JBI) were chosen [10], and to assess the methodological quality of the studies, the norms of Effective Public Health Practice Project (EPHPP) were chosen (Table 2).

## 3. Results

The data collected on the characteristics of the studies were synthesized in a table (Table 3), facilitating mapping. The information in the table is complemented with a narrative summary, in which the findings of the studies included are clarified with the discussion of the results and the description of their correlation with the objective of the review and research question.

All articles selected in this Systematic Literature Review are studies published between 2014 and 2018, as journal articles, with five of these articles being observational descriptive studies and one article being a quasi-experimental study. They carefully present their aims, where they took place, the methods used, and their participants.

Regarding the place where the studies were performed, two were performed in the United States of America (USA), two in Switzerland, one in Sweden, and one in Australia.

All studies considered participants who were attending high school, so the results of different articles were obtained through self-report analysis of the adolescents, namely through interview. One of the articles analyzed [4] associated this method with an evaluation of sleep behavior using a motor activity recording device during sleep.

The analyzed studies evaluate, in general, the relation between the use of electronic devices and adolescents’ sleep, with special emphasis on the use of smartphones in five of these articles [3,12,13,14,15].

Johansson et al. [3] reported that about 47% of the respondents used three or four devices before going to bed, and 97% used some type of technology in the hour before bedtime, in particular, the mobile phone (used by nearly 74% of respondents). They also concluded that the number of devices used before bedtime would be associated with reports of waking up too early and restless sleep, with repercussions in daytime sleepiness.

In fact, the use of mobile phones, specifically smartphones, is highlighted by several authors. Schweizer et al. [12] verified that smartphone owners were more likely to experience sleep problems than non-smartphone owners, who usually sleep the number of hours recommended by the National Sleep Foundation. They observed that as soon as the adolescents owned a smartphone, they would start to sleep less than what is recommended, increasing the prevalence of sleep problems to achieve the prevalence observed among owners.

Lemola et al. [15] also concluded that having a smartphone would be related to a greater use of electronic devices and, in particular, their use in bed, before sleep, by watching videos, phone calling, and texting with friends. The use of these electronic devices in bed before sleep was associated with less sleep time and difficulty in sleeping, such as being online in bed and with the phone turned on at night, which would intensify these changes.

Tavernier et al. [4] verified that adolescents sleep less when they spend more time than usual texting and working on the computer, and even that more time using the computer was associated with less sleep efficiency. On the contrary, they verified a positive association between the time spent in direct social contact (with friends and family) and the efficiency and latency of sleep; that is, when adolescents spend more time interacting directly with friends/family, they fall asleep faster.

Likewise, Garmy and Ward [13] highlighted the nighttime use of mobile phones, associating sending and receiving messages at night with much later hours to fall asleep, less regular hours to fall asleep, shorter times in bed, and irregular sleep habits. The authors also verified that tiredness during the day was frequently associated with this. Like these authors, Vernon et al. [14] also established a significative correlation between the nighttime use of phones, bad sleep behavior, and a decline in adolescents’ well-being. The authors verified that the nighttime use of the phone would is also associated with depressive humor, low self-esteem, externalizing behavior, and low coping ability.

## 4. Discussion

Adolescence is a period of great physical, emotional, and social changes, in which sleep is susceptible to modification inherent to these changes. Variations in bedtime hour, waking hour, and sleep habits are very common among adolescents, with sleep disorders considered a public health problem in the United States of America, Europe, and Asian countries [13].

Technological development has been growing exponentially in the last few decades and, consequently, the use of electronic devices now plays an important role in adolescents’ lives [13,14]. With the current systematic literature review, it was intended to understand the relation between the use of new technologies, particularly smartphones, and the sleep of adolescents. The studies analyzed corroborate the existing literature, reinforcing the fact that the use of technological gadgets influences the sleep of adolescents [3,4,12,13,14,15]. Among electronic devices, the smartphone stands out for its use, disseminated especially among adolescents, its ease of access, and its functionalities [12,14].

Schweizer et al. [11] verified that the owners of a smartphone were more prone to having sleep problems than non-owners, who slept, on average, for the number of hours recommended by the National Sleep Foundation. They observed that as soon as the adolescents owned a smartphone, they would start to sleep less than recommended, increasing the prevalence of sleep problems to achieve the prevalence observed among owners. The authors state that smartphones enable Internet access anywhere at any time, in addition to allowing communication through texts messages and free phone calls, which justifies the increase in possession of smartphones among adolescents.

Tavernier et al. [4] verified that adolescents sleep less when they spend more time than usual using electronic-based devices, such as texting and working on a computer, and, furthermore, more time using a computer was associated with reduced sleep efficiency. On the contrary, they verified a positive association between the time spent in direct social contact (with friends and family) and the efficiency and latency of sleep, meaning when adolescents spend more time than usual interacting directly with friends/family, they fall asleep faster.

The constant connectivity in technological devices has been a risk factor for good-quality adolescent sleep, as already confirmed in the literature [16]. The elevated quantity of time spent by adolescents with technological-based activities is undeniable, usage that is also verified at nighttime, namely the hour before bedtime, so several studies have focused on its use in this period. Lemola et al. [15] concluded that the possession of a smartphone would be related to a generally greater use of electronic devices, and, in particular, the use in bed, before sleep, watching videos, for phone calls, or texting friends.

If, on the one hand, the screen time stands out, on the other hand, there seems to be an association between owning a smartphone and the use of electronic devices at night.

The high amount of time spent by adolescents on technology-based activities is undeniable, with studies suggesting that this directly influences sleep time and efficiency. 

In fact, Johansson et al. [3] verified that 97% of adolescents used some type of technology in the hour before bedtime (especially the mobile phone, used by nearly 74% of respondents). Further, 47% of the respondents used three or four devices in this time period. Vernon et al. [14] also corroborated this idea by concluding that there were few adolescents that did not use the mobile phone during nighttime after switching off the lights to sleep. 

The studies reviewed suggest that there is a relationship between screen time and sleep, especially when used at night. Johansson et al. [3] verified the existence of a direct relation between the use of electronic devices and the sleep changes in adolescents, with its use before bedtime being associated with reports of waking up too early, restless sleep, and daytime sleepiness. This association was also verified by Vernon et al. [14] and by Lemola et al. [15], who highlighted that being online in bed and with the phone turned on at night would exacerbate these changes. Likewise, Garmy and Ward [13] highlighted the nighttime use of mobile phones, associating the sending and receiving of messages at night with much later hours to fall asleep, less regular hours to fall asleep, shorter times in bed, and irregular sleep habits. The authors also verified that frequently associated with this, there would be tiredness during the day. 

Like these authors, Vernon et al. [14] also established a significative correlation between the nighttime use of phones and bad sleep behavior.

According to the literature, this influence can be explained, on the one hand, by the cognitive, emotional, and physiological excitement associated with social interactions and videogaming at night and, on the other, by the light exposure emitted by screens, which can suppress the secretions of melatonin, contributing equally to difficulties in falling asleep [3,13,14].

Many authors established the relation between sleep problems associated with the use of new technologies and the well-being and psychosocial behavior of adolescents. Vernon et al. [14] established a significative correlation among nighttime use of phones, bad sleep behavior, and the decline in adolescents’ well-being. The authors verified that nighttime use of the phone would also be associated with depressive humor, low self-esteem, externalizing behavior, and low coping ability.

Lemola et al. [15] also concluded that the use of smartphones may be a partial mediator of the relation between the use of these electronic devices at night and depressive symptoms. They also established a relation regarding sleep, depressive symptoms, and the use of electronic devices before sleep, which meets the existing literature on the subject: “Recent studies show a link between insufficient sleep and mental health problems, including anxiety depression, low self-esteem and suicidality”; at the same time, it has negative effects on academic performance and overall performance during the day [13] (p.123). This phenomenon seems to occur, according to the author, because adolescents manifest feelings of concern and disconnection when unable to access technology, fearing the loss of information or important messages.

In Portugal, in 2017, a study was conducted about epidemiology of internet use in a population of adolescents and its relationship with sleep habits, published in Portuguese Medical minutes [17], in which 722 adolescents were inquired for establishing a parallel between use of the internet and, consequentially, the associated technology (mobile phones, online gaming devices, computers, tablets) and sleep habits. The study was based on the premise that the adolescents’ use of internet and its excessive use were associated with dysfunctional coping strategies and worsen interpersonal relationships, negative impact on psychosocial development of adolescents, its association with suicidal behaviors (suicidal ideation and suicide attempts), depression, and anxiety. Therefore, the authors intended to “measure and characterize the use of internet in adolescence; determine the internet dependence on adolescence; evaluate the association between internet dependency and sleep changes/excessive daytime tiredness” [17] (p. 52).

Meeting the results of the findings in the present review, the study revealed that the internet dependence and its problematic use could have a significant influence on the sleep–wake cycle associated with intermediate insomnia, irregular sleep patterns, and excessive daytime tiredness, which was mentioned by Garmy and Ward [13]. It also tried to explain that the “negative impact of excessive internet use and sleep habits could be due to the use of devices (computer/mobile phone/tablet) while falling asleep, which conditions a state of excitement, therefore, interfering with the necessary procedures of a normal sleep-wake cycle” [17] (p. 531).

This study was the only one found about this topic for this age group in Portugal; it supports the analyzed studies of the present systematic literature review.

Therefore, although the mobile phone is a tool for social interaction that cultivates self-esteem, autonomy, and identity in adolescents, its excessive use, notably at night-time, constitutes a problem, with an impact on the health of adolescents [14]. Thus, the parents/caregivers and the current policies have a preponderant role in the sensibilization of adolescents regarding the disadvantages of nighttime use of technologies/use of mobile phones in the sleep quality of adolescents.

Nursing has a privileged position in the contact with adolescents and families in their daily practice. Therefore, according to Garmy and Ward [13], nurses are in an ideal position to educate adolescents about the importance of healthy and consistent sleep habits and to alert them to the hazards of bad or insufficient sleep habits. According to the same authors, more than the adolescents, “School nurses are also in a unique position to discuss sleep health and educate not only students but also teachers and parents.” (p. 124).

Johansson et al. [3] also considered that the results of the study have implications for nursing care practices: “Nurses are in the ideal position to address their goal by educating adolescents, their families, and the broader community about good sleep hygiene” (p. 502). The author also reinforces the importance of nurses in primary health care, as “Nurses in primary care or outpatient clinics should inquire about an adolescent’s sleep habits, sleep quality, and technology use before bed. In addition, nurses should take the opportunity to counsel families in healthy sleep habits.” (p. 502), meeting what was previously stated by Garmy and Ward [13].

In this sense, measures were suggested that are focused on the healthy use of technology in the beginning of adolescence, informing the adolescents and their parents that the acquisition of smartphone or other electronic devices can bring consequences regarding sleep quality, which repercuss in the adolescent’s quality of life [3,13].

It is urgent to alert and study, alongside parents, strategies that limit the use of electronic devices, mainly during the nighttime period as a way of promoting quality sleep.

## 5. Conclusions

It was possible to find evidence that confirms the influence of technological devices, such as smartphones, computers, and tablets, in the sleep of adolescents. The results of the present systematic literature review suggest that this influence may be mainly associated with screen time. More studies are needed that explore the relation between adolescent sleep and screen time, even during the day. 

The influence of technology, highlighting the use of smartphones, repercusses both in terms of quality and sleep duration, with manifestations in the performance of adolescents during the day and their well-being.

It becomes urgent to promote the implementation of measures that encourage a healthy use of new technologies, specifically at night.

Based on scientific assumptions, the nurse, in collaboration with other members of the multidisciplinary team, alongside adolescents, parents, and educators, could develop interventions that promote the thoughtful use of these resources, as this approach should be developed in the follow-up appointments of infant and juvenile health. Despite the present systematic literature review focusing on adolescents, the approach towards a healthy education for the use of this resource should be initiated as soon as possible. 

## 6. Limitations/Future Prospects

Limitations were identified in some of the studies found, especially regarding the age of the study participants, which is not consensual, and it is not always identified for the authors to present only the year of schooling. Some of the results were also based on adolescents’ self-report and, in some cases, obtained through questionnaires conducted in class, which may condition the adolescents’ answers and their veracity. In the future, research will be needed that considers all these factors. 

## Figures and Tables

**Table 1:** Critical evaluation of the results of the included studies according to the critical evaluation verification list by Joanna Briggs Institute [11].

Articles	Q1	Q2	Q3	Q4	Q5	Q6	Q7	Q8	Q9	Q10	Result
Tavernier et al. [4]	✓	✓	✓	✓	✓	✕	✓	✓	-	-	87.5%
Johansson et al. [3]	✓	✓	✓	✓	✓	✓	✓	✓	-	-	100%
Schweizer et al. [12]	✓	✓	✓	✓	✓	✓	✓	✓		-	100%
Garmy & Warde [13]	✓	✓	✓	✓	✓	✓	✓	✓	-	-	100%
Vernon et al. [14]	✓	✓	✓	✓	✓	✓	✕	✓	✓	✓	90%
Lemola et al. [15]	✓	✓	✓	✓	✓	✕	✓	✓	-		87.5%

**Table 2:** Classification of the selected studies according to evidence and methodological recommendation level.

Article	Level of Evidence JBI [11]	Methodological Recommendation (EPHPP)
Tavernier et al. [4]	4.b	87.5%
Johansson et al. [3]	4.b	100%
Schweizer et al. [12]	2.a	100%
Garmy and Warde [13]	4.b	100%
Vernon et al. [14]	4.c	90%
Lemola et al. [15]	4.b	87.5%

**Table 3:** Extraction data.

Study Identification	Country	Method	Study Objective	Total of Participants	Intervention
Tavernier et al., (2017) [4]	USA	Analytical Cross-Sectional Study	To analyze the influence of use of time by the adolescents (in a technological, and face-to-face interactions with friends and family, activity base) in three sleep behaviors: time to fall asleep, duration and sleep efficiency.	71 students of 3 High School, from 11 to 18 years old	The participants used a device, in the non-dominant wrist, that registered the motor activity, for three nights, after a night of “familiarization” as a software and its algorithm were used to register data. The participants filled out a questionnaire about the time spent with eight activities of technological, and face to face interaction with friends and family, activity base.
			To understand the impact of technology use before bedtime on the sleep of adolescents and its repercussion the following day.	255 adolescents between 13 and 21 years old	Data obtained from a conducted study (National Sleep Foundation’s 2011 Sleep in America Poll) with American population between 13 and 64 years old. A subsample was selected that answered a questionnaire developed by a group of experts in the field of sleep. The questionnaire had four categories: demographic data, sleep habits, quality of sleep and the use of technology one hour before bedtime and throughout the night.
Schweizer et al., (2017) [12]	Switzerland	Quasi-Experimental Study	To evaluate the influence of acquiring a smartphone in the sleep duration of adolescents.	591 students of 35 High Schools with an average age of 14.2 years old	The sample was grouped into owners of a smartphone, new owners, and non-owners. The initial collection was done through a questionnaire in classroom. The remaining data were collected through an online questionnaire.
Garmy & Ward, (2018) [13]	Sweden	Analytical Cross-Sectional Study	To understand the relation between the use of the mobile phone in the pre-sleep period and the sleep habits of adolescents.	278 students of 3 High Schools, with ages between 15 and 17 years old	A pre-test was applied to the students, following a session on health education about sleep and usage of social networks after which the test was repeated.
Vernon et al., (2018) [14]	Australia	Case Serie Study	Analyze the relation between the use of a mobile phone in night-time and the quality of sleep and well-being of adolescents.	1101 students, of 29 schools, who began Year 8 in high school	The data were extracted through a longitudinal study; Study based on data of self-report for four consecutive years (2010–2013), beginning in Year 8; All students were submitted to a data collection for at least 2 times.
Lemola et al., (2014) [15]	Switzerland	Analytical Cross-Sectional Study	To evaluate the relation between the use of electronic devices before bedtime and the possession of a smartphone; To understand the relation between sleep, depressive symptoms, and the use of electronic devices at night.	362 students at High School with ages between 12 and 17 years old.	The participants filled out a questionnaire about sleep, media consumption before sleep and psychological health. After completing the questionnaire, the students received information about sleep hygiene or general information about the topics related to sleep. One month after, the students were visited a second time and completed the same questionnaire.

## Data Availability

Not applicable.
